# Immunoproteasome Activity in Chronic Lymphocytic Leukemia as a Target of the Immunoproteasome-Selective Inhibitors

**DOI:** 10.3390/cells11050838

**Published:** 2022-03-01

**Authors:** Andrej Besse, Marianne Kraus, Max Mendez-Lopez, Elmer Maurits, Herman S. Overkleeft, Christoph Driessen, Lenka Besse

**Affiliations:** 1 Laboratory of Experimental Oncology, Department of Oncology and Hematology, Cantonal Hospital St. Gallen, 9000 St. Gallen, Switzerland; andrej.besse@kssg.ch (A.B.); marianne.kraus@kssg.ch (M.K.); max.mendezlopez@kssg.ch (M.M.-L.); christoph.driessen@kssg.ch (C.D.); 2 Gorlaeus Laboratories, Leiden Institute of Chemistry, 2333 CC Leiden, The Netherlands; e.maurits@lic.leidenuniv.nl (E.M.); h.s.overkleeft@lic.leidenuniv.nl (H.S.O.)

**Keywords:** immunoproteasome, chronic lymphocytic leukemia, acute myeloid leukemia, multiple myeloma, plasma cell leukemia, proteasome inhibitors, LU005i, LU035i, activity-based probes, proteasome activity

## Abstract

Targeting proteasome with proteasome inhibitors (PIs) is an approved treatment strategy in multiple myeloma that has also been explored pre-clinically and clinically in other hematological malignancies. The approved PIs target both the constitutive and the immunoproteasome, the latter being present predominantly in cells of lymphoid origin. Therapeutic targeting of the immunoproteasome in cells with sole immunoproteasome activity may be selectively cytotoxic in malignant cells, while sparing the non-lymphoid tissues from the on-target PIs toxicity. Using activity-based probes to assess the proteasome activity profile and correlating it with the cytotoxicity assays, we identified B-cell chronic lymphocytic leukemia (B-CLL) to express predominantly immunoproteasome activity, which is associated with high sensitivity to approved proteasome inhibitors and, more importantly, to the immunoproteasome selective inhibitors LU005i and LU035i, targeting all immunoproteasome active subunits or only the immunoproteasome β5i, respectively. At the same time, LU102, a proteasome β2 inhibitor, sensitized B-CLL or immunoproteasome inhibitor-inherently resistant primary cells of acute myeloid leukemia, B-cell acute lymphoblastic leukemia, multiple myeloma and plasma cell leukemia to low doses of LU035i. The immunoproteasome thus represents a novel therapeutic target, which warrants further testing with clinical stage immunoproteasome inhibitors in monotherapy or in combinations.

## 1. Introduction

The differentiation of human B-cells from their progenitors to immunoglobulin-secreting cells is completed in a series of clearly recognized, discrete stages. At each step of the B-cell differentiation, a cell can undergo malignant transformation, thus giving rise to various malignancies arising from the B-cell lineage. Poorly differentiated acute B-cell malignancies, such as B-cell acute lymphoblastic leukemia (B-ALL) are more common in children than in adults, while the most prevalent B-cell malignancies in adults are chronic lymphocytic leukemia (B-CLL) or malignancies of terminally differentiated plasma cells, such as multiple myeloma (MM) or plasma-cell leukemia (PCL). While B-CLL is the most common chronic type of leukemia in adults, the most common type of acute leukemia in adults is acute myeloid leukemia (AML), a malignancy arising from a myeloid progenitor (National Cancer Institute: https://seer.cancer.gov accessed on 20 January 2022).

The treatment of these hematological malignancies has considerably improved in the past years with the recent approval of several novel agents for the treatment of AML, B-ALL, B-CLL and MM, which contributed to expanding the palette of therapeutic options in these diseases [1,2,3,4]. However, the main reason for treatment failure in a significant proportion of adult patients with leukemias or MM is the occurrence of intrinsic or acquired drug resistance in a subset of malignant cells that is responsible for the development of relapse or refractory disease with a dismal prognosis [5,6,7,8]. Subsequently, as the development of drug resistance is one of the limiting factors affecting long-term efficacy of anti-leukemic or anti-myeloma drugs, the search for therapies with novel mechanisms of action is an ongoing challenge.

Proteasome inhibitors (PIs), such as boronate-based bortezomib and ixazomib and epoxyketone-based carfilzomib specifically inhibit proteasomes, which are large protein complexes with three main catalytic subunits β1, β2 and β5, providing the proteasome with caspase-like, trypsin-like and chymotrypsin-like activities to digest and recycle ubiquitin-tagged proteins [9]. By design, PIs bind to the active pocket of the proteasome β5 subunit, which was initially identified as a rate-limiting protease for functional proteasomal degradation. Only recently, the importance of other proteasome subunits has been shown. The proteasome β5 subunit allosterically activates the β1 subunit [9,10,11], but its co-inhibition has not shown a strong additional cytotoxic effect, whereas the functional β2 subunit co-inhibition together with β5 inhibition is cytotoxic in MM and breast cancer cells [12,13,14].

PIs are cornerstones of treatment of plasma cell malignancies, such as MM, PCL, and mantle cell lymphoma [15]. Moreover, they were extensively evaluated for the therapy of other myeloid or lymphoid malignancies. Although the pre-clinical data showed efficacy of PIs bortezomib and carfilzomib in B-CLL [16,17,18,19], AML [20,21] and ALL [22], clinical observations of PIs in monotherapy in B-CLL, AML or ALL [23,24,25,26] did not fully confirm this data as most of the patients experienced only modest anti-leukemic activity of PIs. Moreover, the patients experienced several toxic side effects from bortezomib therapy, whereas carfilzomib was rather well tolerated.

In the cells of hematopoietic origin, the constitutive proteasome is replaced by the immunoproteasome, in which the standard β1, β2 and β5 catalytic subunits are replaced by the inducible subunits β1i (LMP2), β2i (MECL-1) and β5i (LMP7) [27]. Immunoproteasome expression is noticeably induced upon stimulation by inflammatory cytokines, such as interferon-γ (IFN-γ) and tumor necrosis factor-α (TNF-α) [28]. The primary function of immunoproteasome is to generate more hydrophobic peptides, which are more likely to be presented by HLA molecules (MHC class I molecules) [29,30], but it also plays an important role in protein homeostasis control. Not only does it regulate quality control and clearance of oxidized proteins and protein aggregates generated under cytokine-induced oxidative stress, but it also controls protein transcription and levels of transcription factors that regulate multiple signaling pathways [31].

Given the abundance of immunoproteasome in several leukemia types or myeloma cells, selective targeting of the immunoproteasome is an attractive treatment option [32]. The recent development of immunoproteasome-specific PI may further allow selective targeting of such increased immunoproteasome activity to overcome drug resistance, while sparing the vast majority of tissues not expressing the immunoproteasome, thus considerably reducing the secondary effects and toxicities related to PI treatment.

To date, the proteasome/immunoproteasome composition and activity in the most common subtypes of adult leukemia is unknown. Moreover, we lack data that compare the activity of individual proteolytic subunits of the constitutive versus the immunoproteasome to the cytotoxic activity of the approved PI or novel immunoproteasome-selective PIs. Therefore, we used a cocktail of activity-based proteasome probes (ABPs), which covalently bind to the proteolytically-active sites of the constitutive and the immunoproteasome in a way that corresponds to their catalytic activity [33], to assess the proteasome content in primary samples of patients with AML, B-CLL, B-ALL, MM and PCL. Subsequently, we related the proteasome activity to the cytotoxicity of bortezomib and carfilzomib and of the novel immunoproteasome selective inhibitors LU005i and LU035i [34]. We identified B-CLL to have exclusively high immunoproteasome activity over the constitutive proteasome activity that may be used as a novel target for the immunoproteasome-selective inhibitors. Moreover, the malignancies inherently resistant to immunoproteasome-selective inhibitors may be sensitized to their cytotoxic activity by the selective inhibition of the proteasome β2 subunit.

## 2. Materials and Methods

### 2.1. Patients’ Samples

Primary samples of patients with B-cell acute lymphoblastic leukemia (ALL), acute myeloid leukemia (AML), chronic B-cell lymphocytic leukemia (CLL), multiple myeloma (MM) and plasma cell leukemia (PCL) and peripheral blood mononuclear cells (PBMC) from healthy volunteers were obtained at the Clinics for Medical Oncology and Hematology, Cantonal Hospital St. Gallen, St. Gallen, Switzerland. All samples were obtained during routine diagnostic procedures after approval by the independent cantonal ethical committee and after obtaining written informed consent form in accordance with Helsinki Declaration guidelines.

B-ALL, AML, B-CLL and PCL samples were obtained from peripheral blood. B-ALL and AML samples were enriched by CD34+ selection (EasySep Human Cord Blood CD34 Positive Selection Kit II, StemCell Technologies, Vancouver, BC, Canada). B-cell CLL samples were isolated using EasySep Direct Human B-CLL Cell Isolation Kit (StemCell Technologies, Vancouver, BC, Canada). MM plasma cells samples were obtained from bone marrow aspirates, enriched by CD138+ selection using EasySep Human Whole Blood and Bone Marrow and CD138+ positive selection kit (StemCell Technologies, Vancouver, BC, Canada). PBMC were obtained by standard Ficoll gradient separation of the peripheral blood.

### 2.2. Cell Culture

Primary cells were thawed into RPMI-1640 medium (Sigma-Aldrich, Buchs, Switzerland) supplemented with 20% heat-inactivated fetal calf serum (FCS), 100 mg/mL streptomycin and 100 U/mL penicillin (all Sigma-Aldrich, Buchs, Switzerland) and seeded for further analyses.

AMO-1 cell line was obtained from commercial sources (American Type Culture Collection, ATCC/LGC, Wesel, Germany). AMO-1 wild-type and AMO-1 PSMB5KO cell lines were maintained under standard conditions in RPMI-1640 medium supplemented with 10% FCS, 100 mg/mL streptomycin and 100 U/mL penicillin. MycoAlert Mycoplasma Detection Kit (Lonza, Basel, Switzerland) was used to rule out the mycoplasma contamination in the cell culture and the modified cell line was authenticated with its parental cell line by the STR-typing (at DSMZ, a German collection of Microorganisms and Cell Cultures, Braunschweig, Germany).

### 2.3. CRISPR/Cas9 Knockout of PSMB5

Two different short guide RNAs (sgRNAs) targeting PSMB5 gene in positions spanning the proteolytically active site were designed by web-based tool CRISPOR [35], to generate a larger deletion in PSMB5 gene, as was described before [36]. The proteolytically active site of human β5c subunit was obtained from UniProt database (P28074). The sequences of the sgRNAs are as follows (with the PAM sequence in italics): PSMB5_g1: CCGCTACCGGTGAACCAGCGC*GGG*, PSMB5_g2: TGCCTCCCAGACGGTGAAGA*AGG*.

Briefly, sgRNAs PSMB5_g1 and PSMB5_g2 were cloned into lentiCRISPRv2 vector plasmid carrying both Cas9 and guide RNA (a gift from Zhang’s lab; Addgene plasmids #52961). Next, separate lentiviruses for PSMB5_g1 and PSMB5_g2 were produced by packaging plasmids pMD2.G and psPAX2 (a gift from Trono’s lab; Addgene plasmids #12259 and #12260) and the lentiCRISPRv2 transfer plasmids in HEK-293-LentiX cells (Takara-Bio, Kusacu, Japan) following protocol described elsewhere [37]. AMO-1 cells were transduced with PSMB5_g1 and PSMB5_g2 viral particles in 1:1 ratio and cells with stably introduced CRISPR/Cas9 vectors were selected by puromycin (2 µg/mL, Sigma-Aldrich, Buchs, Switzerland). Single-cell derived colonies of AMO-1 cells were obtained using MethoCult Classic (#H4434, StemCell Technologies, Vancouver, BC, Canada). The clones were screened for larger deletions in PSMB5 using PCR with primers spanning the deletion site (PSMB5_del_F: AGGAAGTGAAGCTGTGACGG and PSMB5_del_R: CGTTCCCAGAAGCTGCAATC) that produces PCR product of 1442 bp in non-deleted PSMB5 and 250 bp in deleted PSMB5. Sanger sequencing was performed to confirm the presence of a deletion in the on-target sequence of genome. Moreover, the clones were screened for knock-out of the β5c activity by ABP and a single-cell derived colony with confirmed deletion and no β5c activity was chosen for further analyses.

### 2.4. Chemicals

Bortezomib and carfilzomib were obtained from commercial sources (Selleck Chemicals, Houston, TX, USA). LU005i, LU035i, LU025c, LU102 and ABP were synthesized at Leiden University. Detailed information regarding proteasome inhibitors used in the study is presented in Table S1.

### 2.5. Proteasome β-Subunits Profiling with Activity-Based Proteasome Probes Labelling

Activity of proteasome subunits was assessed on a protein lysate by SDS-PAGE after 1 h/37 °C incubation with the set of subunit-selective activity-based probes (ABP) that differentially visualize individual activities of β1, β2 and β5 subunits of the constitutive and the immunoproteasome, as described [33]. Protein subunits were separated by SDS-PAGE, gel images were acquired using Fusion Solo S Western Blot and Chemi Imaging System (Vilber Lourmat, Collégien, France). The quantification of the activity was performed using Fiji (open source image processing package based on ImageJ) [38]. For each sample, the ratio of activity of the immunoproteasome vs. constitutive proteasome subunits was calculated by dividing the band intensity of each of the immunoproteasome subunits by the band intensity of the corresponding constitutive proteasome subunit.

### 2.6. CTG Viability Assay

An amount of 1 × 10^4^ of cells were seeded per well into a white, flat bottom 96-well plate (Corning, Root, Switzerland). The cells were exposed to increasing doses of proteasome inhibitors in 100 µL of standard media per well for 48 h and cell viability was determined using CellTiter-Glo luminescent cell viability assay (Promega, Madison, WI, USA) according to manufacturer’s protocol. Only samples where the untreated controls showed high ATP production were used in the analysis. The cytotoxicity of the drugs was normalized to control—untreated cells—and for each sample a dose-response curve to each tested chemical was generated.

### 2.7. Lactate Dehydrogenase Quantification

Levels of lactate dehydrogenase (LDH, assessed in U/L) from peripheral blood of the patients were determined during patients’ routine diagnostic procedures at the Cantonal Hospital, St. Gallen.

### 2.8. Statistical Analysis

Dose-response curves were generated using nonlinear fit. The IC_50_ of each chemical was determined using nonlinear regression analysis from dose-response curves. Ordinary one-way ANOVA with Tukey’s multiple comparisons test was used for the comparison of statistically significant differences between the samples. Correlation coefficients between the activity ratios of β5i/c, the cytotoxicity of proteasome inhibitors and LDH levels were calculated using Spearman’s rank correlation, and *p* values < 0.05 were considered as statistically significant. Statistical evaluation was performed in GraphPad Prism v8 (GraphPad Software, La Jolla, CA, USA).

## 3. Results

### 3.1. B-CLL Shows Exclusive Predominant Activity of the Immunoproteasome

Initially, the activity of both types of proteasomes were tested in different hematological malignancies, including 16 AML, 3 B-ALL, 17 B-CLL, 6 MM and 5 PCL and in PBMC samples obtained from six healthy donors. Detailed characteristics of patients/samples included in the study is provided in Table 1.

**Figure 1:**
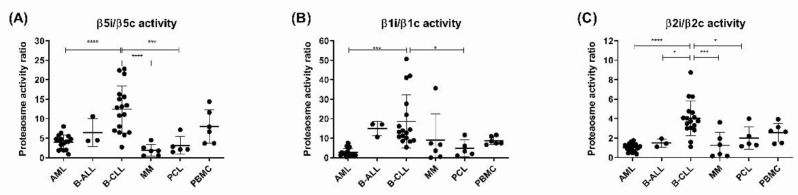
Profile of the activity of the immunoproteasome subunits over constitutive proteasome subunits determined by ABP labelling in different hematological malignancies. (**A**) Comparison between the ratio of activity of proteasome β5i versus β5c, data represent mean ± SD. (**B**) Comparison between the ratio of activity of proteasome β1i versus β1c, data represent mean ± SD. (**C**) Comparison between the ratio of activity of proteasome β2i versus β2c, data represent mean ± SD. In all analyses, statistical significance was obtained with ANOVA and Tukey’s multiple comparison test, where * represents *p* < 0.05, *** represents *p* < 0.001 and **** represents *p* < 0.0001. AML = acute myeloid leukemia, B-ALL = B-cell acute lymphoblastic leukemia, B-CLL = B-cell chronic lymphocytic leukemia, MM = multiple myeloma, PCL = plasma-cell leukemia, PBMC = peripheral blood mononuclear cells.

In each sample, activity of each of the proteolytically active β-subunits was determined by ABP and is expressed as a ratio between the respective immunoproteasome and the constitutive proteasome subunit (β5i/c, β1i/c and β2i/c). While most of the malignancies show activity of both types of the proteasomes, B-CLL samples show increased activity of the immunoproteasome active sites β5i, β1i and β2i over the corresponding β5c, β1c and β2c sites (Figure 1A–C). The most significant differences were observed in the activity ratios for β5 and β2 subunits, where B-CLL differed significantly in β5i/c activity ratio from AML, T-ALL, MM and PCL (Figure 1A) and in β2i/c activity ratio from AML, B-ALL, MM and PCL (Figure 1C). Deeper analysis of B-CLL cohort of patients (for basic biological and clinical information about the B-CLL cases, see Supplementary Table S2) showed that levels of lactate dehydrogenase (LDH) correlate positively with the β5i/β5c activity ratio (Spearman *r* = 0.6818; *p* = 0.0251, Supplementary Figure S1A). Of note, PBMC samples show rather heterogeneous activity profile of the β5i/β5c, as they are a mixture of different cell types with different proteasome activities (as, for example, normal B-cells predominantly express β5i) [39]. Nevertheless, from the malignant entities of a B-cell origin, B-CLL shows a unique profile of high relative immunoproteasome activity, which is not seen in other acute or chronic B-cell malignancies and which correlates with levels of LDH, a general marker of tumor burden.

### 3.2. B-CLL Is the Most Sensitive to Bortezomib and Carfilzomib

**Figure 2:**
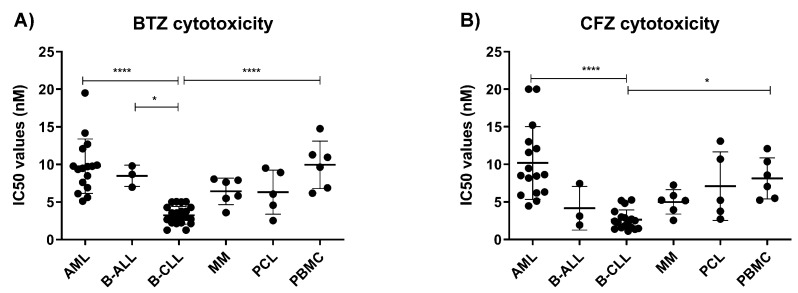
Profile of the IC_50_ values of the approved proteasome inhibitors in different hematological malignancies. (**A**) Comparison of IC_50_ values of bortezomib determined 48 h after the continuous treatment in various hematological malignancies, data represent mean ± SD. (**B**) Comparison of IC_50_ values of carfilzomib determined 48 h after the continuous treatment in various hematological malignancies, data represent mean ± SD. In all analyses, statistical significance was obtained with ANOVA and Tukey’s multiple comparison test, where * represents *p* < 0.05 and **** represents *p* < 0.0001. AML = acute myeloid leukemia, B-ALL = B-cell acute lymphoblastic leukemia, B-CLL = B-cell chronic lymphocytic leukemia, MM = multiple myeloma, PCL = plasma-cell leukemia, PBMC = peripheral blood mononuclear cells, BTZ = bortezomib; CFZ = carfilzomib.

The approved PIs for MM therapy are designed to target the chymotrypsin-like site (β5 subunit) of the constitutive and immunoproteasome, which is the most important target to inhibit proteasomal proteolysis [40,41]. It was later discovered that higher doses of bortezomib co-inhibit caspase-like sites (the β1 subunits), while carfilzomib co-inhibits trypsin-like sites (the β2 subunits). Moreover, they target the active sites of the immunoproteasome at low nanomolar doses, comparable to doses necessary for the inhibition of the active sites of the constitutive proteasome [42,43,44]. Therefore, we tested the cytotoxicity of bortezomib and carfilzomib in our cohort of hematological malignancies by analyzing dose-response curves and obtaining IC_50_ value for each sample. B-CLL was the most sensitive cohort of samples to both bortezomib and carfilzomib, supporting our previous observations (Figure 2A,B). Specifically, B-CLL samples were significantly more sensitive to bortezomib and carfilzomib than AML, which was the most resistant cohort of samples in our analysis. Importantly, only B-CLL cells were also significantly more sensitive to bortezomib and carfilzomib than PBMCs, showing an opportunity for a selective toxicity of malignant cells and less systemic toxicity associated with the use of these PIs.

Nevertheless, although B-CLL is uniformly sensitive to bortezomib and carfilzomib, there was no correlation between sensitivity of the individual samples to bortezomib or carfilzomib and their β5i/c activity ratio. This suggests that B-CLL depend on functional proteasome activity, irrespective of its type.

### 3.3. Immunoproteasome-Selective Proteasome Inhibitors Are Selectively Cytotoxic in B-CLL and Their Cytotoxicity Correlates with Immunoproteasome Activity

**Figure 3:**
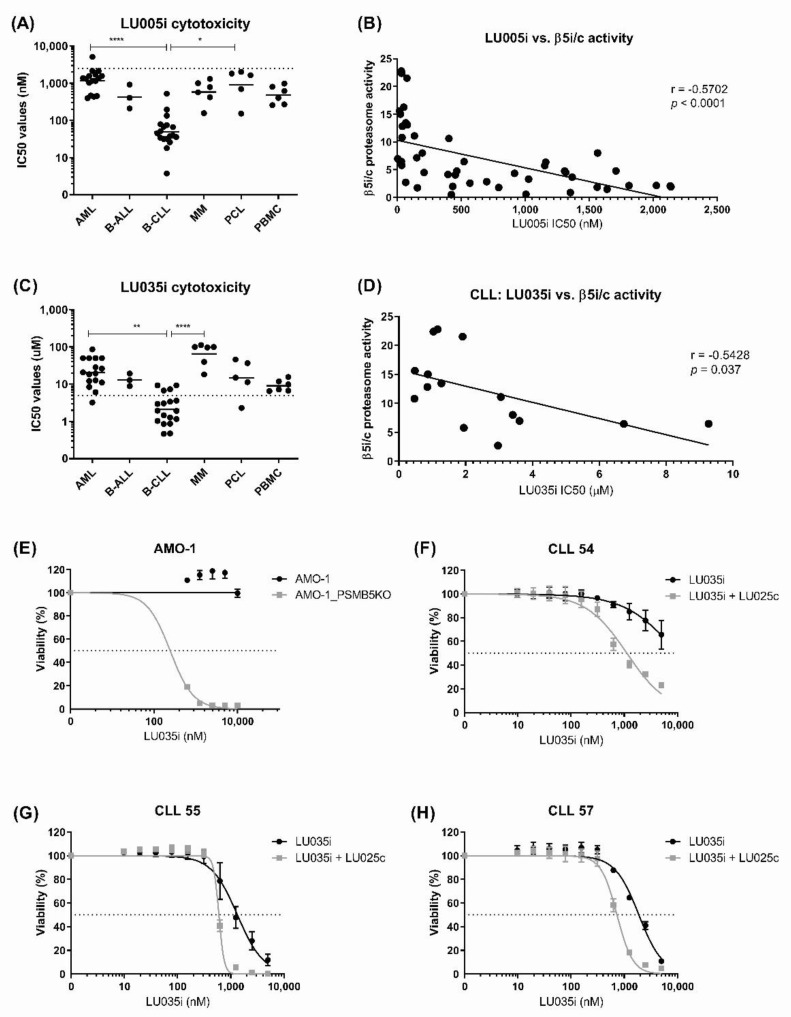
Cytotoxicity of the immunoproteasome-selective proteasome inhibitors in hematological malignancies. (**A**) Comparison of IC_50_ values of LU005i determined 48 h after the continuous treatment in various hematological malignancies. Data represent geometric mean ± geometric SD, statistical significance was obtained with ANOVA and Tukey’s multiple comparison test, where * represents *p* < 0.05 and **** represents *p* < 0.0001. Line represents a 2.5 µM dose, to which the inhibitor retains its selectivity. (**B**) Correlation between the activities of constitutive vs. the immunoproteasome β5 subunits and the cytotoxicity of LU005i in 15 AML, 3 B-ALL, 17 CLL, 6 MM and 5 PCL samples. Correlation and statistical significance were obtained using Spearman’s rank correlation. (**C**) Comparison of IC_50_ values of LU035i determined 48 h after the continuous treatment in various hematological malignancies, data represent geometric mean ± geometric SD. In samples, where the IC_50_ value was not reached, it was arbitrarily given an IC_50_ = 100 µM. Statistical significance was obtained with ANOVA and Tukey’s multiple comparison test, where ** represents *p* < 0.01 and **** represents *p* < 0.0001. Line represents a 5 µM dose, to which the inhibitor retains its selectivity. (**D**) Correlation between the activities of constitutive vs. the immunoproteasome β5 subunits and the cytotoxicity of LU035i in 17 CLL samples. Correlation and statistical significance were obtained using Spearman’s rank correlation. (**E**) Dose-response curves of AMO-1 and AMO-1 PSMB5 knock-out cells to LU035i determined 48 h after the treatment. Data represent mean ± SD of three independent experiments. (**F**–**H**) Dose-response curves of three B-CLL samples to LU035i alone or in combination with 1 µM LU025c determined 48 h after the treatment. Data represent mean ± SD of tetraplicate. AML = acute myeloid leukemia, B-ALL = B-cell acute lymphoblastic leukemia, B-CLL = B-cell chronic lymphocytic leukemia, MM = multiple myeloma, PCL = plasma-cell leukemia, PBMC = peripheral blood mononuclear cells; LU005i = proteasome β5i + β2i + β1i selective inhibitor; LU035i = proteasome β5i selective inhibitor; LU025c = proteasome β5c selective inhibitor; i = immunoproteasome, c = constitutive proteasome.

Since B-CLL shows the highest relative immunoproteasome activity, it could be exclusively sensitive to novel selective immunoproteasome inhibitors. These inhibitors could preserve efficacy on malignant cells, but significantly reduce treatment-emergent toxicities by sparing other tissues with little to no immunoproteasome activity [32]. First, we tested the cytotoxic activity of novel immunoproteasome inhibitors on a cohort of various hematological malignancies. We chose LU005i for selective inhibition of the immunoproteasome active subunits β5i, β1i and β2i with predominant activity on β5i > β1i > β2i at low micromolar doses [34] (Supplementary Figure S2). LU005i was cytotoxic in low micromolar range (below 2.5 µM, where it retains the selectivity for the immunoproteasome subunits) in all tested hematological malignancies. Moreover, it was significantly more cytotoxic in B-CLL cells with high relative immunoproteasome activity, in contrast to AML or PCL, which keep both types of active proteasomes (Figure 3A). Cytotoxicity of LU005i correlated with the activity ratios of the β5i/c subunit across the whole cohort of hematological malignancies tested (Figure 3B); however, we did not observe any significant correlation between the activity ratios of individual β subunits and cytotoxicity of LU005i in B-CLL.

The chymotrypsin-like site of the immunoproteasome (β5i) is more hydrophobic and has different structure and size than the chymotrypsin-like site of the constitutive proteasome (β5c) [45]. Therefore, it allows the design of selective β5i inhibitors. Since β5i is rate-limiting for the proteolytic activity of the immunoproteasome, as is the β5c for the activity of the constitutive proteasome, we hypothesized that the sole inhibition of the β5i subunit activity with LU035i may be sufficient to induce cytotoxicity in cells with predominant immunoproteasome activity. LU035i is an epoxyketone-based selective PI that is selective for the inhibition of the β5i subunit up to 5 µM concentration [34] (Supplementary Figure S3). Almost all B-CLL samples were sensitive to cytotoxic activity of LU035i at low micromolar doses (Figure 3C), in contrast to the AML, B-ALL, MM and PCL samples, suggesting that β5i may be a novel therapeutic target in B-CLL. Moreover, the cytotoxicity of LU035i correlated with the β5i/c activity ratios in B-CLL (Figure 3D), whereas it could not be properly assessed in other malignancies, since the inhibitor did not reach the IC_50_ values here or else reached it at very high doses, where it most likely loses the β5i selectivity. At the same time, as we observed a positive correlation between β5i/β5c activity ratios and levels of LDH, we likewise observed here a negative correlation between LDH levels and IC_50_ values of LU035i (Supplementary Figure S1B, Spearman *r* = −0.6545, *p* = 0.0336).

Previously, we have shown that in cells with both β5i and β5c activities, inhibition of β5i is not cytotoxic, as the residual β5c activity can substitute for inhibited β5i activity [12]. Since we observed the correlation between β5i/c activity ratios and cytotoxicity of LU035i in B-CLL, we aimed to assess if the cytotoxicity of LU035i is solely related to predominant β5i activity or also to other factors. At the same time, we aimed to assess to what extent a presence of β5i only, or both β5i and β5c activities, affects the cytotoxicity of LU035i. Towards this aim, we first knocked-out the β5c activity in AMO-1 MM cell line by introducing a deletion in *PSMB5*, including the enzymatic active site. We subsequently obtained a single-cell derived colony with no detectable β5c activity, but with present β5i activity (Supplementary Figure S4). At the same time, we chemically inhibited the residual β5c activity with selective β5c inhibitor LU025c [46] in B-CLL samples, which showed lower cytotoxicity of LU035i. As expected, LU035i was not cytotoxic in AMO-1 wild-type cells up to 10 µM concentration, whereas it was cytotoxic in AMO-1_PSMB5KO cells at sub micromolar doses (Figure 3E). Likewise, inhibition of residual β5c with LU025c sensitized B-CLL cells to LU035i (Figure 3F–H). Of note, AMO-1_PSMB5KO cells completely lacking the active β5c were not sensitized to LU035i by LU025c up to 3 µM concentration, whereas AMO-1 wt were sensitized significantly (Supplementary Figure S5A,B), confirming that the residual β5c activity is the only factor affecting cytotoxicity of LU035i in malignant cells. Therefore, high relative activity of the β5i subunit is a signature of B-CLL representing a novel therapeutic target for immunoproteasome β5-selective inhibitor LU035i, associated with low cytotoxicity on cells with low relative immunoproteasome activity.

### 3.4. β2-Selective Proteasome Inhibitor Sensitizes Hematological Malignancies to β5i-Selective Immunoproteasome Inhibitor

**Figure 4:**
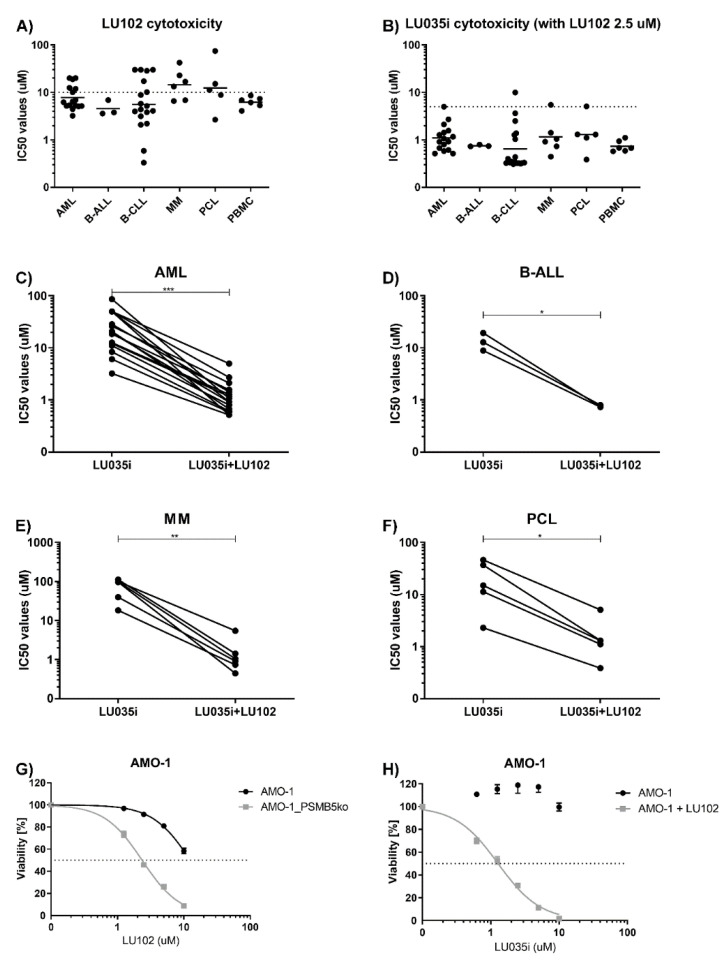
β2-selective inhibitor sensitizes hematological malignancies to immunoproteasome-selective inhibitors. (**A**) Comparison of IC_50_ values of LU102 determined 48 h after the continuous treatment in various hematological malignancies, data represent geometric mean ± geometric SD. Statistical significance was obtained with ANOVA and Tukey’s multiple comparison test. (**B**) Comparison of IC_50_ values of LU035i combined with fixed dose of LU102 (2.5 µM) determined 48 h after the continuous treatment in various hematological malignancies. Data represent geometric mean ± geometric SD. Statistical significance was obtained with ANOVA and Tukey’s multiple comparison test. (**C**) Paired comparison of IC_50_ values of LU035i combined with fixed dose of LU102 (2.5 µM) determined 48 h after the continuous treatment in AML samples. Statistical significance was obtained with paired *t*-test, where *** represents *p* < 0.001. (**D**) Paired comparison of IC_50_ values of LU035i combined with fixed dose of LU102 (2.5 µM) determined 48 h after the continuous treatment in B-ALL samples. Statistical significance was obtained with paired *t*-test, where * represents *p* < 0.05. (**E**) Paired comparison of IC_50_ values of LU035i combined with fixed dose of LU102 (2.5 µM) determined 48 h after the continuous treatment in MM samples. Statistical significance was obtained with paired *t*-test, where ** represents *p* < 0.01. (**F**) Paired comparison of IC_50_ values of LU035i combined with fixed dose of LU102 (2.5 µM) determined 48 h after the continuous treatment in MM samples. Statistical significance was obtained with paired *t*-test, where * represents *p* < 0.05. (**G**) Dose-response curves of AMO-1 wt and AMO-1 *PSMB5* knock-out cells to LU102 determined 48 h after the treatment. Data represent mean ± SD. (**H**) Dose-response curves of AMO-1 cells to LU035i alone or in combinations with 2.5 µM LU102, determined 48 h after the treatment. Data represent mean ± SD. AML = acute myeloid leukemia, B-ALL = B-cell acute lymphoblastic leukemia, B-CLL = B-cell chronic lymphocytic leukemia, MM = multiple myeloma, PCL = plasma-cell leukemia, PBMC = peripheral blood mononuclear cells; LU035i = proteasome β5i selective inhibitor; LU102 = proteasome β2c and β2i selective inhibitor; i = immunoproteasome, c = constitutive proteasome.

Co-inhibition of the proteasome β2c and β2i activity sensitizes MM cells to immunoproteasome inhibitor ONX-0914 [47]. Thus, we assessed if samples inherently not sensitive to β5i inhibition may be sensitized to LU035i by co-inhibition of the β2c and β2i activity with selective inhibitor LU102 [48]. In living cells, LU102 sub-totally inhibits both β2c and β2i activity at 10 µM dose and retains its β2 selectivity up to 20 µM [12]. We have not observed any difference in the cytotoxicity of LU102 across different malignancies (Figure 4A). However, 2.5 µM LU102, a dose not affecting the viability in most of the samples, significantly sensitized all tested hematological malignancies to low doses of LU035i, where it retains selectivity only for the β5i inhibition (Figure 4B–F). Moreover, LU102 alone was more cytotoxic in AMO-1_PSMB5KO cells, as compared to AMO-1 wild-type cells (Figure 4G), suggesting that lower drug doses are needed to induce cytotoxicity in the absence of β5c activity. This aligns with previous data, where in the presence of β5i and β5c inhibition, lower doses of LU102 were needed to achieve complete β2i and β2c inhibition [12]. At the same time, 2.5 µM dose of LU102, not affecting the viability of AMO-1 wild-type cells, significantly sensitized the cells to β5i-selective immunoproteasome inhibitor LU035i (Figure 4H). Therefore, combination of the immunoproteasome selective inhibitors with β2c and β2i selective inhibitor allows using lower doses of both drugs to induce cytotoxicity in hematological malignancies, such as AML, MM and PCL.

## 4. Discussion

Targeting immunoproteasome is a treatment strategy clinically tested in autoimmune diseases and experimentally explored in pediatric ALL or adult malignancies, such as MM [47,49,50,51]. Here we show that B-CLL cells possess increased activity of the immunoproteasome subunits over the constitutive proteasome active subunits, which is associated with their high sensitivity to approved PIs bortezomib and carfilzomib or novel immunoproteasome-selective inhibitors LU005i and LU035i. While bortezomib and carfilzomib inhibit both the constitutive and the immunoproteasome, they have been shown unlikely to move forward in B-CLL as a single agent given the minimal efficacy observed. At the same time, high selectivity and cytotoxic activity of LU035i in B-CLL, and significant correlation between the cytotoxicity and the immunoproteasome β5i activity assessed by ABP labelling suggests that proteasome inhibition is still interesting to pursue as part of combination strategies in which efficacy of another drug may be improved by inhibition of proteasome-mediated protein breakdown. Moreover, proteasome activity assessment should be used for patients’ stratification and identification of patients that may benefit the most from such therapy.

The molecular mechanism underlying high immunoproteasome activity in B-CLL remains to be elucidated. The activity of the immunoproteasome can be reversibly stimulated by pro-inflammatory cytokines IFN-γ or TNF-α, or by reactive oxygen species (ROS) [52,53,54,55], which may potentially influence the composition of proteasome in B-CLL; however, multiple other factors may be involved. At the same time, as we observed a positive correlation between high relative β5i/β5c activity and levels of LDH, it suggests that higher relative immunoproteasome activity is associated with more severe disease and poorer prognosis [56]. Increased LDH levels are associated with poor prognosis in myelodysplastic syndromes, AML and B-CLL [57,58,59]. It remains to be elucidated whether immunoproteasome inhibition decreases LDH levels, a potential useful marker of disease control.

Currently, two immunoproteasome inhibitors have entered clinical evaluation for the treatment of autoimmune disorders. Both, ONX-0914 and KZR-616, are selective irreversible inhibitors of β5i (LMP7) and β1i (LMP2) sites of the immunoproteasome [60,61]. Following the discovery of their activity against autoimmune disorders, their anti-tumor activities were tested in selected groups of patients with leukemia. ONX-0914 showed activity in pediatric ALL, in contrast to rather low activity in pediatric AML [49]. More recently, ONX-0914 has been shown to be effective in pediatric T-ALL cases with t (4; 11) (q21; q23) chromosomal translocation that leads to the expression of MLL–AF4 fusion protein conferring poor outcome [50]. Newly, orally bioavailable reversible immunoproteasome β5i inhibitor M3258 showed efficacy in diverse in vitro and in vivo MM models, a favorable safety profile and a lack of cardiac, respiratory, and neurobehavioral effects, supporting the initiation of a phase I clinical trial of M3258 in patients with relapsed/refractory MM (NCT04075721) [62,63]. In this study, we tested LU005i and LU035i irreversible immunoproteasome inhibitors, which selectively target immunoproteasome subunits over a broad concentration range up to micromolar doses [34]. Our data extend previous observations and shows that a sole inhibition of the β5i subunit in B-CLL is sufficient to induce cytotoxicity, which proportionally corresponds to the high relative immunoproteasome β5i activity ratio. At the same time, healthy PBMCs are less vulnerable to LU035i-induced cytotoxicity, thereby offering a therapeutic window at which cells expressing both types of proteasomes are spared from on-target toxicity of LU035i.

Previous data suggested that β2 inhibition ex vivo sensitizes adult malignancies, such as MM, or pediatric B-ALL and T-ALL cases to ONX-0914 or LU035i [47,49,50]. Our data support these results and show that β2 inhibition provided by LU102 sensitizes B-CLL cells to β5i inhibition provided by LU035i. More importantly, malignancies with the activity of both types of proteasomes, such as AML, B-ALL, MM or PCL, which are intrinsically resistant to LU035i, can be sensitized to its cytotoxicity by LU102. These findings offer novel therapeutic possibility for the treatment of malignancies with lack of effective therapies.

To the best of our knowledge, this is the first study showing composition and activity of proteasome/immunoproteasome in the most common adult hematological malignancies and demonstrating the exclusive activity of the immunoproteasome-selective inhibitors in B-CLL. We acknowledge the limitation of our study, which is the low number samples used in the analysis that does not allow further stratification of patients to major cytogenetically defined subgroups with different prognoses. We observed consistently low activity ratios between the constitutive vs. the immunoproteasome β-subunits in AML, MM and PCL, whereas in B-CLL, the activity ratios varied considerably from high to intermediate/low. These observations require further studies on larger cohorts of patients.

## 5. Conclusions

In conclusion, we provide a strong rationale for further in vivo studies of immunoproteasome-selective inhibitors in B-CLL, which may provide higher activity and lower off-target toxicity in combination setting with other drugs used for B-CLL therapy. At the same time, combination of immunoproteasome-selective and β2-selective inhibitors may be effective in hematological malignancies with poor prognosis and lack of effective therapies, such as AML or PCL.

## Figures and Tables

**Table 1:** Basic characteristics of patients included in the study.

	AML	B-ALL	B-CLL	MM	PCL
Nr of patients	16	3	17	6	5
Male-females (%)	62–38%	33–67%	65–35%	50–50%	20–80%
Age (median; min–max)	64 (32–84)	35 (28–38)	69 (54–81)	74 (56–84)	60 (51–69)

AML = acute myeloid leukemia, B-ALL = B-cell acute lymphoblastic leukemia, B-CLL = B-cell chronic lymphocytic leukemia, MM = multiple myeloma, PCL = plasma-cell leukemia.

## Data Availability

There were no data deposited in publicly available data-repositories within this pro-ject. However, any data generated within this project are available upon request.

## Supplementary Materials

The following are available online at https://www.mdpi.com/article/10.3390/cells11050838/s1, Figure S1: Correlation of LDH with proteasome activity and cytotoxicity of the immunoproteasome selective inhibitor in B-CLL primary samples. Figure S2: Inhibitory profile of pan-immunoproteasome selective inhibitor LU005i. Figure S3: Inhibitory profile of β5-immunoproteasome selective inhibitor LU035i. Figure S4: Profile of active proteasome β-subunits in AMO-1 wild-type cells and in AMO-1 cells with *PSMB5* knock-out. Figure S5: Dose-response curves of AMO-1wild-type and AMO-1_PSMB5 knock-out cells to β5i inhibition in a presence or absence of the β5c inhibition. Table S1: Detailed characteristics of proteasome inhibitors used in the study. Table S2: Basic biological and clinical characteristics of B-CLL cohort.
