# Supplementary material for: Immunoproteasome Activity in Chronic Lymphocytic Leukemia as a Target of the Immunoproteasome-Selective Inhibitors
Source: Cells. 2022 Mar 1;11(5):838. doi: 10.3390/cells11050838 (PMC8909520; doi:10.3390/cells11050838)
Supplement: Supplementary file 1 [file cells-11-00838-s001.zip › cells-1594087-supplementary.pdf]

## Supplementary data

### *Supplementary Tables*

**Table S1: Detailed characteristics of proteasome inhibitors used in the study**.

i.v. = intravenous infusion, s.c. = subcutaneous administration

| Proteasome inhibitor | Chemical entity | Route of administration | Clinical Status | Target in the proteasome complex |
|---|---|---|---|---|
| Bortezomib | boronate | i.v. infusion or s.c. | Approved for MM | β5i and β5c (in higher dose β1i and β1c) |
| Carfilzomib | epoxyketone | i.v. infusion | Approved for MM | β5i and β5c (in higher dose β2i and β2c) |
| LU005i | epoxyketone | i.v. | preclinical | β5i, β2i and β1i [34] |
| LU035i | epoxyketone | i.v. | preclinical | β5i [34] |
| LU102 | vinylsulfone | i.v. | preclinical | β2i and β2c [48] |
| LU025c | epoxyketone | i.v. | preclinical | β5c [46] |

**Table S2: Basic biological and clinical characteristics of B-CLL cohort.**

| | **B-CLL** |
|---|---|
| **Nr of patients** | 17 |
| **Sex: male-female (%)** | 11 (65%) - 8 (35%) |
| **Age (median; min-max)** | 69 (54-81) |
| **Sample characteristics** | |
| diagnosis (%) | 1 (5.9%) |
| relapse (%) | 16 (94.1%) |
| **Cytogenetic aberrations** | |
| TP53: del/mut/normal/NA | 3/3/7/4 |
| ATM: del/normal/NA | 3/8/6 |
| 13q14: del/normal/NA | 8/3/6 |
| IGHV status: mutated/unmutated/NA | 3/7/7 |
| **Biochemical characteristics** | |
| Hemoglobin, g/l (median; min-max) | 125.5 (95-144) |
| Leukocytes, g/l (median; min-max) | 81.9 (19-334) |
| Bilirubin, µmol/l (median; min-max) | 9.5 (5-34) |
| LDH, U/l (median; min-max) | 319 (252-622) |

NA = non available; del = deletion; mut = mutation

***Supplementary Figures***

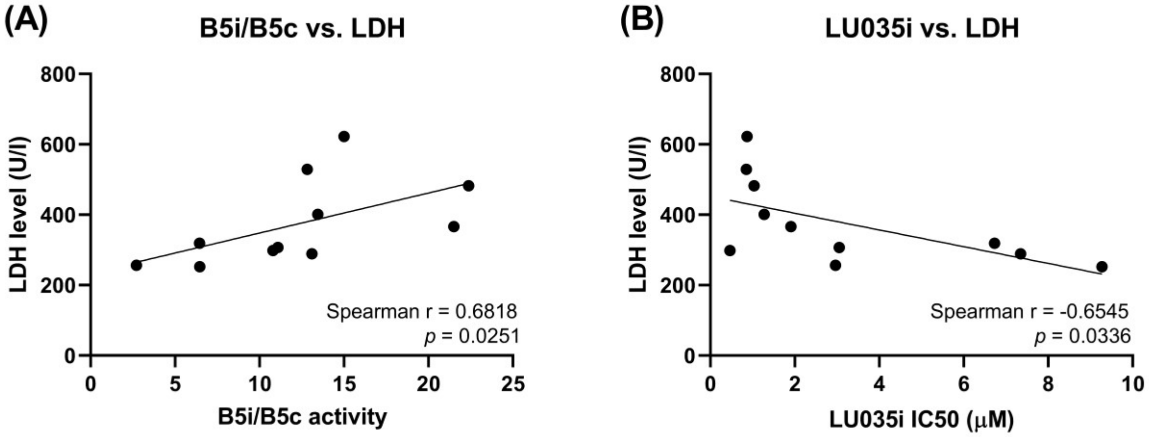


**Figure S1: Correlation of LDH with proteasome activity and cytotoxicity of the immunoproteasome selective inhibitor in B-CLL primary samples. A)** Correlation of LDH with the activity ratio of proteasome β5i/β5c. **B)** Correlation of LDH with the cytotoxicity of LU035i, an immunoproteasome β5i selective inhibitor (presented as the $IC_{50}$ values). Spearman correlation coefficient and *p* value are presented for bot analyses.

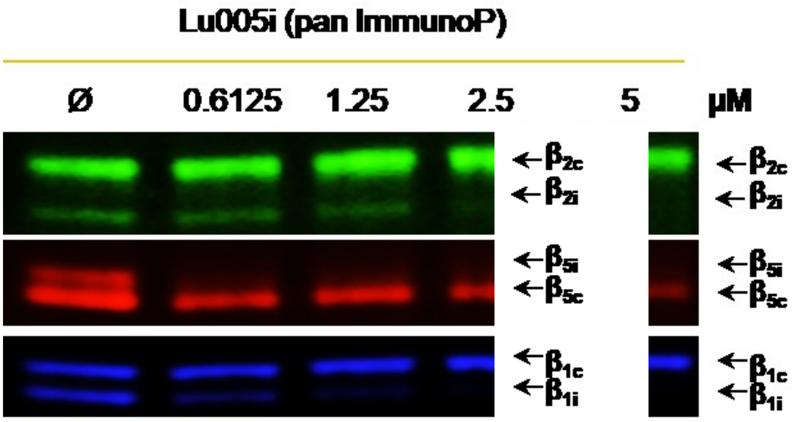


**Figure S2: Inhibitory profile of pan-immunoproteasome selective inhibitor LU005i**. AMO-1 cells were exposed to indicated doses of LU005i for 1h, then lysed and labelled with ABP labelling for 1h to visualize active proteasome β-subunits. Subsequently, the proteins were separated by SDS-PAGE.

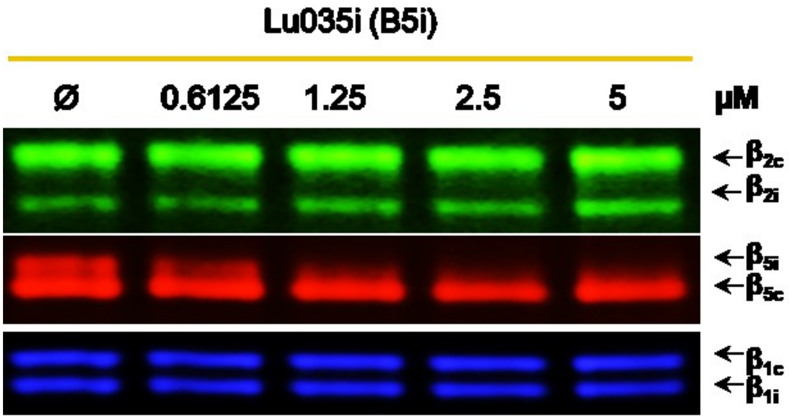


**Figure S3: Inhibitory profile of β5-immunoproteasome selective inhibitor LU035i.** AMO-1 cells were exposed to indicated doses of LU035i for 1h, then lysed and labelled with ABP labelling for 1h to visualize active proteasome β-subunits. Subsequently, the proteins were separated by SDS-PAGE.

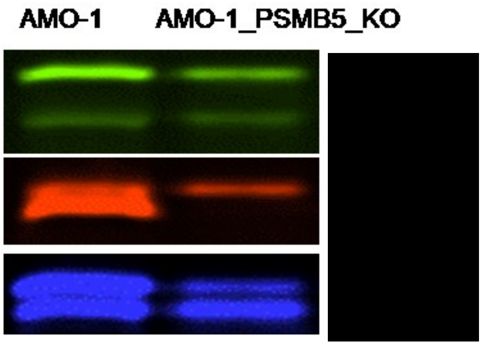


**Figure S4: Profile of proteasome β-subunits activity in AMO-1 wild-type cells and in AMO-1 cells with PSMB5 knock-out.** Cells were lysed and labelled with ABP labelling for 1h to visualize active proteasome β-subunits. Subsequently, the proteins were separated by SDS-PAGE.

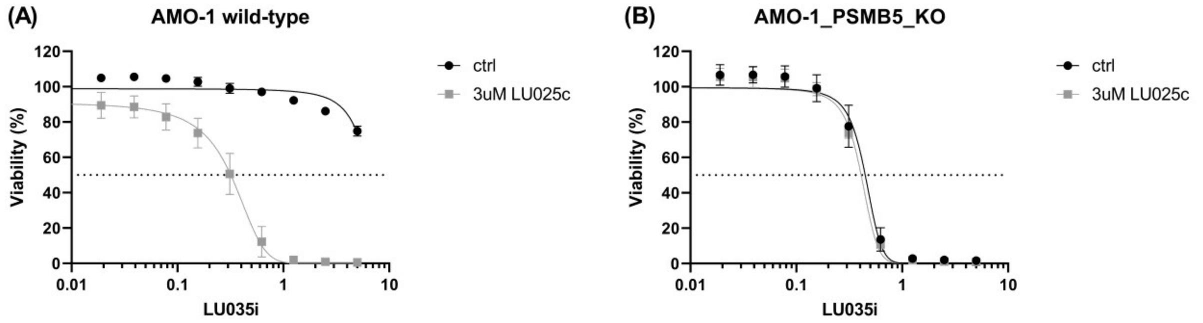


**Figure S5: Dose-response curves of AMO-1 wild-type and AMO-1_PSMB5 knock-out cells to β5i inhibition in a presence or absence of the β5c inhibition.** A) AMO-1wild-type and B) AMO-1_PSMB5_KO cells were exposed to increasing doses of LU035i in monotherapy or co-treated with 3 μM LU025c, viability was determined 48h after the treatment. Data represent mean ± SD of three repeats. LU035i = β5i selective inhibitor, LU025c = β5c selective inhibitor.
